# Medication Patterns as a Lens on Health Needs Among Migrant Agricultural Workers in Informal Settlements in Apulia: A Descriptive Outreach Study

**DOI:** 10.3390/idr18040076

**Published:** 2026-07-21

**Authors:** Cesare De Virgilio Suglia, Renato Laforgia, Marcella Schiavone, Anna Belfiore, Giacomo Guido, Rosa Buonamassa, Alba Cuxart-Graell, Martina Di Noto, Valeria Mele, Emanuele Costanza, Venilia Cocco, Alexandre Meduri, Lucia Raho, Nicole Laforgia, Roberta Iatta, Giovanni Putoto, Francesco Di Gennaro

**Affiliations:** 1 Doctors with Africa CUAMM, 70121 Bari, Italyveni.cocco@gmail.com (V.C.); a.meduri@phd.uniba.it (A.M.); n.laforgia@cuamm.org (N.L.); 2 Internal Medicine “A. Murri”, Department of Precision and Regenerative Medicine and Ionian Area (DiMePRe-J), Polyclinic Hospital, 70124 Bari, Italy; 3 Clinic of Infectious Diseases, Department of Precision and Regenerative Medicine and Ionian Area (DiMePRe-J), University of Bari ‘A. Moro’, 70124 Bari, Italy; 4 Barcelona Institute for Global Health (ISGlobal), 08036 Barcelona, Spain; alba.cuxart@isglobal.org; 5 Interdisciplinary Department of Medicine, University of Bari, 70124 Bari, Italy; roberta.iatta@uniba.it; 6 Operational Research Unit, Doctors with Africa CUAMM, 35121 Padua, Italy; g.putoto@cuamm.org

**Keywords:** migrant health, agricultural workers, public health, structural vulnerability, social determinants, prescribing patterns, labour exploitation, primary health care, embodiment, health inequities

## Abstract

**Background:** Migrant agricultural workers in Italy often experience social and health vulnerabilities, including unstable housing and limited access to primary care. In Southern Italy, many live in informal settlements and seek care through outreach services. This study describes patterns of pharmacological treatment in this population and examines how they relate to the clinical conditions managed in mobile clinics. **Methods:** We analyzed routinely collected data from 2928 unique patients (8547 clinical encounters; 8965 treatment occurrences) managed by Doctors with Africa CUAMM mobile clinics in 12 informal settlements in Apulia, Italy, between 2017 and 2026. Diagnoses were grouped into clinical categories, and pharmacological treatments were classified by therapeutic class. We conducted a descriptive analysis of the distribution of diagnostic categories and associated treatments. **Results:** The population was predominantly male (96.5%), young (81% <45 years), and largely excluded from regular primary care (93.5% without a General Practitioner). Musculoskeletal disorders and fatigue were the leading diagnostic category (34.0%), followed by gastrointestinal (13.5%) and respiratory conditions (13.0%). Non-steroidal anti-inflammatory drugs (NSAIDs) and analgesics were the most frequently recorded treatments (32.6% of treatment occurrences). Among musculoskeletal presentations, NSAIDs were used in 76% of cases. Gastroprotective agents were documented in 52% of encounters with gastrointestinal symptoms. Among cardiovascular presentations, 87.7% of treatment occurrences involved chronic management with antihypertensives or beta-blockers. **Conclusions:** In this outreach setting, medication use provides a descriptive picture of common health problems and treatment responses among migrant agricultural workers living in informal settlements. The prominent use of symptomatic pharmacological relief, particularly NSAIDs in musculoskeletal conditions, suggests that care is often focused on managing pain and acute complaints in a population facing barriers to continuous primary care. These findings support the need for stronger inclusion of migrant workers in the National Health Service and for policies that address underlying social and structural determinants of health.

## 1. Introduction

The Italian agricultural sector relies heavily on migrant labour. Current estimates indicate that approximately 500,000 migrant workers are employed in the sector, and a substantial proportion experience severe exploitation, insecure employment, informal recruitment practices, and poor living conditions [1,2]. In Apulia, one of the main agricultural regions in Southern Italy, many workers live in informal settlements or so-called ‘ghettos’, often in overcrowded and unsafe shelters lacking running water, electricity, sanitation, and adequate protection from heat or cold [3,4,5]. These conditions are closely linked to the caporalato system, occupational insecurity, social exclusion, and limited legal protection, all of which shape both health risk and healthcare access [6,7].

In this population, disease cannot be understood only in biomedical terms. Work intensity, unstable income, fear of job loss, transportation barriers, legal precarity, and substandard housing directly influence when people seek care, which symptoms become prioritized, and whether treatment can realistically be continued over time. As a result, the health needs of migrant agricultural workers often emerge in compressed and highly pragmatic forms: pain must be controlled rapidly, respiratory and dermatological complaints are managed in the field, gastrointestinal symptoms are treated despite limited diagnostic depth, and chronic conditions may remain invisible until outreach services create an opportunity for recognition [1,3,4,7].

Although Italy has a universal healthcare system that formally guarantees the right to essential and urgent care to all individuals regardless of their legal status, access to care remains highly unequal for migrant agricultural workers. Administrative barriers, language and cultural obstacles, limited health literacy, geographic isolation, fear related to legal status, and constant seasonal mobility can all disrupt continuity of care [1,3,4,7]. Even when entitlement to healthcare formally exists (Undocumented migrants can access the healthcare system through the STP code), effective access may still be interrupted by lack of documentation, poor knowledge of services, difficulties in booking appointments, inability to leave work, and a fragile relationship with institutions. In practice, this means that many common and potentially manageable conditions are first encountered late, intermittently, or only when they have already become symptomatic enough to compromise daily functioning.

Since 2017, Doctors with Africa CUAMM has attempted to reduce these barriers through the Supreme project, providing mobile-clinic outreach in 12 informal settlements in Apulia. This model, which combines clinical care with social-health orientation, cultural mediation, basic diagnostics, and referral support, has also been recognized by the World Health Organization as an example of good practice in hard-to-reach migrant populations [8,9]. The outreach setting is therefore not only a place of care delivery, but also an observatory from which the interaction between disease, working conditions, and barriers to the National Health Service can be examined in a particularly concrete way.

Building on this experience, the present study focuses on pharmacological treatment patterns among migrant agricultural workers in Apulia. Rather than describing diagnoses alone, we examined which medicines were prescribed across different clinical conditions and considered these patterns as possible markers of working and living hardship, unmet needs, and restricted access to standard care. This perspective is relevant because medicines in outreach healthcare services are not neutral items: they may indicate whether care is predominantly symptom-oriented, whether definitive treatment pathways are lacking, and whether chronic conditions are being intercepted only partially.

By analyzing the distribution of therapeutic classes and their relationship with major diagnostic categories, we sought to provide a more specific understanding of how structural determinants of health are translated into everyday therapeutic practice in this setting. In particular, we aimed to assess whether prescribing trends could help illuminate the practical consequences of labour exploitation, housing precarity, environmental exposure, and exclusion from routine primary care among migrant agricultural workers living in Southern Italy.

## 2. Materials and Methods

### 2.1. Study Design and Objectives

The primary objective of this study was to describe the pharmacological profile of care delivered by mobile clinics in informal settlements. A secondary objective was to examine how major therapeutic classes were distributed across the most frequent clinical presentations, in order to identify recurrent treatment patterns in a highly marginalized population.

This study was designed as a retrospective descriptive analysis of routinely collected outreach data from mobile clinics operated by Doctors with Africa CUAMM in informal settlements in the Apulia countryside, Southern Italy, between June 2017 and March 2026. The present analysis specifically focused on pharmacological treatments delivered during outreach activities and on their distribution across the main diagnostic categories.

### 2.2. Study Setting and Outreach Model

The study setting consisted of 12 geographically isolated informal settlements inhabited mainly by migrant agricultural workers living in conditions of marked social and material precarity. Most residents lived in makeshift shelters built from sheet metal and recycled materials, often without reliable access to electricity, safe water, sanitation, cooling systems, or adequate winter protection. Exposure to heat, cold, humidity, crowding, and occupational contaminants was therefore a recurrent feature of life in the settlements.

To reach this population, Doctors with Africa CUAMM implemented a mobile clinic model aimed at reducing the practical and symbolic distance between patients and services. Outreach activities were conducted inside or in close proximity to the settlements, minimizing transportation barriers and facilitating access for people who might otherwise postpone or forgo care. Within the constraints of a field setting, the service integrated symptom management, first-line clinical evaluation, selected diagnostic activities, referral to higher levels of care, and social-health orientation.

The outreach team was multidisciplinary and included physicians, nurses, medical residents from different specialties, cultural mediators, and social workers. The mobile clinic was equipped with essential medicines, point-of-care diagnostic tools, and selected rapid tests, including HIV, HCV, and COVID-19 screening. On average, the team carried out eight outreach sessions per month, adapting activities to seasonal agricultural cycles. Clinical management in the field included immediate symptomatic treatment when necessary, prescription of medications to be obtained through the health system, and direct provision of drugs available on site, as well as wound care, health education, and referral to emergency departments, specialist clinics, or territorial services when conditions required further evaluation.

### 2.3. Study Population and Recruitment

The target population consisted of migrant agricultural workers residing in the 12 informal settlements. All individuals living in these settlements who voluntarily sought care at the mobile clinic during the study period were invited to participate and were eligible for inclusion. There were no specific exclusion criteria other than the absence of complete diagnostic or treatment data in the records.

Participants were recruited through active outreach within the settlements, facilitated by cultural mediators who explained the voluntary nature of the service. The analysis therefore reflects clinical encounters and treatment events among users of the outreach service, rather than all migrant workers living in the settlements.

### 2.4. Data Collection

Clinical and demographic data were systematically recorded in a dedicated Microsoft Access database (Microsoft Corporation, Redmond, WA, USA). Variables available for the present analysis included age, sex, area of origin, selected indicators of administrative inclusion (such as possession of a residence permit and registration with a General Practitioner), presenting clinical category, and pharmacological treatment delivered during the encounter.

For the purposes of this study, we used the term “pharmacological treatment” to encompass three modalities: medications prescribed for subsequent dispensing through the National Health Service, medications administered directly during the outreach visit, and medications physically provided to patients from the mobile clinic’s stock. All three modalities were documented in the database and were treated as part of the pharmacological profile of care.

Before analysis, the database was reviewed for internal consistency and duplicate entries. When repeated records referred to the same individual, patients were counted once in patient-level summaries, while all eligible clinical presentations and treatment occurrences were retained for event-level descriptive analyses. Records lacking either diagnostic classification or treatment information were excluded from the analytical dataset. No imputation procedures were applied, as the study was designed as a descriptive analysis of routinely collected outreach data.

### 2.5. Diagnostic and Pharmacological Classification

Diagnoses were grouped into broad clinical categories reflecting the principal reason for encounter, such as musculoskeletal and fatigue-related conditions, gastrointestinal disorders, respiratory diseases, dermatological conditions, cardiovascular and metabolic disorders, and other less frequent categories.

Pharmacological interventions were grouped into major therapeutic classes, including NSAIDs and analgesics, topical treatments and dressings, adjuvants and supplements, gastroenterological drugs, respiratory drugs, cardiovascular and metabolic treatments, and antibiotics or systemic antifungals. Where relevant, medications were additionally coded by therapeutic subgroup and ATC classification to improve pharmacoepidemiological interpretability.

Diagnostic and therapeutic categories were created through pragmatic aggregation of routinely recorded field data in order to ensure clinical interpretability and consistency across the study period. Individual diagnoses were assigned to the category that best reflected the principal reason for encounter, whereas medications were classified according to their main therapeutic use in the outreach setting. This approach was intended to reduce fragmentation of heterogeneous field records and to allow a meaningful description of recurrent care needs, although some overlap between clinical domains is inherently possible in mobile-clinic practice.

### 2.6. Definition of Treatment Occurrences

For analytic clarity, we defined a “treatment occurrence” as each documented instance in which at least one pharmacological intervention was recorded during a clinical encounter. When more than one medication was prescribed, administered, or provided in the same encounter, each medication was counted as a separate treatment occurrence within the corresponding therapeutic class. This allowed us to describe both the number of encounters involving pharmacological activity and the distribution of individual medications and classes across diagnostic categories.

### 2.7. Data Analysis

All analyses were descriptive. Categorical variables were summarized as absolute frequencies and percentages. To avoid conflating person-level and encounter-level information, the dataset was analyzed at three distinct levels. First, demographic and administrative variables such as age, nationality, legal status, and General Practitioner registration were summarized at the patient level, focusing on the 2928 unique individuals in the cohort. Second, we conducted an encounter-level analysis to characterize the broader clinical profile, reflecting the total burden of the 8547 recorded clinical presentations. Third, pharmacological activity was summarized at the treatment level, covering all 8965 registered treatment occurrences.

This multi-level approach was adopted because a single clinical encounter in an outreach setting can generate multiple diagnostic labels and several treatment entries. Consequently, these counts were treated as discrete care events rather than mutually exclusive person-level outcomes, in order to more accurately represent the clinical workload and the complexity of therapeutic responses provided in the field.

Finally, cross-tabulations were used to examine the distribution of major therapeutic classes across clinical categories and to present, by diagnostic subgroup, the most frequent active ingredients, therapeutic subgroups, and ATC codes, together with absolute case numbers and percentage shares within each subgroup. As the study aimed to provide an exploratory and descriptive account of an underserved population, no formal hypothesis testing, multivariable modelling, or causal inference was performed.

## 3. Results

### 3.1. Study Population

The study population was predominantly composed of young adult men living in conditions of marked social exclusion. Among 2928 unique patients, 96.5% were male, with the largest age groups being 30–45 years (56.5%) and under 29 years (24.4%). Most patients (75%) originated from West and North African countries. Only 18.6% had a residence permit, and 93.5% were not registered with a General Practitioner, indicating substantial exclusion from routine healthcare access.

These baseline characteristics are important for interpreting the therapeutic profile described below. The cohort does not represent a generic primary-care population, but rather a mostly young, highly mobile workforce with limited formal integration into the health system.

### 3.2. Clinical Findings and Morbidity Patterns

A total of 8547 clinical presentations were recorded across the study period. The main diagnostic categories are summarized in Table 1.

Musculoskeletal complaints and fatigue were the most frequent reasons for consultation, accounting for 34.0% of all visits. Gastrointestinal disorders represented 13.5% of presentations, and respiratory conditions 13.0%.

Dermatological conditions accounted for 11.0% of presentations, while dental problems represented 6.5%. Cardiovascular and metabolic conditions were recorded in 8.8% of encounters. A small number of severe conditions, including seven neoplasms, were also identified during outreach activities. In these cases, patients were referred to specialists and hospital services for further management.

The average number of visits per patient was 2.9 during the study period.

### 3.3. Pharmacological Treatment Patterns

#### 3.3.1. Distribution of Therapeutic Classes

We examined pharmacological treatments recorded during outreach activities. Table 2 summarizes the main therapeutic classes documented in the dataset.

Across 8965 treatment occurrences, NSAIDs and analgesics were the most frequently recorded drugs, accounting for 32.6% of all treatments. Topical treatments represented 14.2%, adjuvants and supplements 12.3%, gastroenterological drugs 8.4%, respiratory drugs 8.0%, cardiovascular and metabolic treatments 7.1%, and antibiotics or systemic antifungals 7.1%. A further 9.8% of entries were classified as “none or diagnostics”, indicating encounters in which no pharmacological therapy was provided or in which the clinical act mainly consisted of assessment, testing, isolation advice, or another non-pharmacological decision.

Overall, the pharmacological profile was dominated by symptomatic and supportive treatments.

#### 3.3.2. Alignment Between Diagnostic Categories and Treatment Patterns

To improve pharmacoepidemiological readability, pharmacological treatments are presented by diagnostic subgroup in tabular form. Table 3 reports, for each clinical category, the main therapeutic subgroups, example ATC codes, absolute numbers of treatments, and percentage shares within the subgroup.

Among patients presenting with fatigue syndrome and musculoskeletal conditions, 76% received NSAIDs or analgesics (Table 3). A further 19% received adjuvants or supplements, while 5% were treated with topical products or local interventions.

Among 1157 patients with digestive complaints, 52% received gastroprotective treatment. Probiotics and supplements were used in 27% of cases, symptomatic gastrointestinal drugs (including laxatives, antiemetics, antispasmodics, and antidiarrheals) in 12%, and antibiotics in 3%.

Respiratory conditions were recorded in 1107 patients (Table 3). Of these, 46% were treated with anti-inflammatory agents, 34% with symptomatic respiratory drugs such as mucolytics, cough suppressants, antihistamines, or bronchodilators, and 12% with antibiotics.

Among 944 patients with dermatological problems (Table 3), 57% received topical treatments, including emollients and protectants, wound and ulcer preparations, topical antipruritics, antimicrobials for dermatological use, and corticosteroid creams. Oral antihistamines were used in 14.2% of cases, oral antifungals in 14%, and oral antibiotics or antivirals in 5.7%.

Among 553 patients with dental complaints, 67% received anti-inflammatory drugs or analgesics and 29% received antibiotics.

Cardiovascular conditions were documented in 651 patients (Table 3). Among these, 87.7% received antihypertensives, beta-blockers, or diuretics, while antiplatelet or anticoagulant therapies were recorded in 6% of cases.

## 4. Discussion

This study provides a focused analysis of pharmacological treatments delivered to migrant agricultural workers living in informal settlements in Apulia. In this outreach setting, prescribing patterns can be viewed as a descriptive lens on the cumulative effects of labour conditions, housing precarity, environmental exposure, and systemic barriers to healthcare, rather than as definitive causal evidence of specific determinants [1,3,4,8,10]. Within this framework, the prominence of NSAIDs and gastroprotective agents suggests a care model largely centred on short-term symptomatic relief in a precarious labour market [11,12].

Across all visits, NSAIDs and analgesics accounted for nearly one third of recorded treatments, with particularly frequent use in musculoskeletal presentations and dental complaints. These patterns are consistent with the physical demands of repetitive manual labour, prolonged standing, heavy lifting, and heat exposure in the agricultural setting [3,4,10]. In such contexts, symptomatic pharmacological management may be one of the few immediately feasible responses to ongoing physical strain. Similar profiles have been described in other precarious labour environments, where presenteeism and delayed care-seeking are shaped by economic insecurity and fear of losing income [11,12,13]. Although our data do not directly measure self-medication or work attendance, the treatment distribution is compatible with a model of care in which acute symptom control often takes precedence over long-term resolution.

The observed burden of gastrointestinal (13.5%) and respiratory (13.0%) conditions further aligns with the social and environmental characteristics of informal settlements [5,10,14,15]. These morbidity patterns are plausibly influenced by irregular nutrition, variable food and water quality, overcrowded living conditions, extreme seasonal temperatures, exposure to smoke or dust, and chronic psychosocial stress [5,10,14,15]. Housing conditions likely play a substantial role in respiratory morbidity: many residents live in poorly insulated shelters that are extremely hot in summer and cold in winter, with limited ventilation and insufficient environmental protection [3,4,10]. Such circumstances facilitate persistent cough, upper and lower respiratory symptoms, and prolonged recovery from common infections. Dermatological diseases can similarly be interpreted in relation to sun exposure, contact with agricultural chemicals, and restricted hygiene opportunities due to the lack of running water.

The management of dental conditions illustrates more clearly the interplay between pharmacological care and structural barriers. Most patients with dental complaints received analgesics or antibiotics, while access to definitive dental procedures remained limited. In practice, medications often appear to substitute for specialist interventions that are difficult to access, a pattern also documented among marginalized migrant workers in other contexts [16]. In this sense, prescribing choices may indirectly reveal the absence or fragility of referral pathways.

The high use of gastroprotective treatment among patients with digestive complaints suggests both clinical and iatrogenic mechanisms. Frequent use of anti-inflammatory drugs, especially in the context of irregular or insufficient food intake, may increase the risk of gastritis and dyspepsia [14,15]. The prominence of gastroprotective prescribing can therefore be interpreted as a response to gastrointestinal symptoms and, at the same time, as an indirect indicator of a therapeutic environment in which pain is heavily treated and acid-related symptoms are anticipated. Gastrointestinal complaints are also likely to reflect broader psychosocial and environmental stressors, including migratory trauma, insecurity, and food insecurity, which influence wellbeing and symptom perception [13,17].

Cardiovascular and metabolic diseases appeared less frequently than acute symptomatic conditions in our dataset, yet the treatment profile suggests that chronic illness may be underestimated in this population. The substantial proportion of cardiovascular presentations requiring antihypertensive or related therapy indicates that clinically significant disease is present and may only become visible when active outreach facilitates diagnosis [18,19,20,21,22]. The very low proportion of diabetes-specific treatment may analogously reflect underdiagnosis, limited screening, or low treatment continuity rather than a true absence of disease [23,24]. These considerations should be interpreted in light of wider discontinuities of care associated with seasonal mobility, administrative barriers, and exclusion from primary care [18].

Taken together, these findings support the notion that pharmacological patterns can function as informative indicators of structural vulnerability. Medicines in this setting are not merely therapeutic choices; they also signal work intensity, environmental exposures, barriers to specialist referral, and limited continuity of care. This interpretation reinforces the need for migrant-sensitive, flexible, and culturally competent health services, as emphasized by international guidance [25,26,27,28,29]. Structural vulnerability frameworks remind us that social and economic forces—such as precarious labour, unstable housing, and restricted access to primary care—are “embodied” as physical symptoms, which then shape both clinical presentations and prescribing decisions [3,4,8,10,27,30].

From a public health perspective, improving health outcomes in this population requires more than proximity-based prescribing. There is an urgent need for earlier access to primary care and more effective inclusion in the National Health Service. The pharmacological profile observed here may be interpreted as a warning signal for policymakers: persistent reliance on symptomatic treatment is unlikely to decrease unless underlying social determinants—including unsafe housing and exploitative labour conditions—are addressed. Trust-building and cultural competence remain essential. As noted by the WHO, healthcare professionals should develop both clinical and structural competence to recognize how social conditions shape disease and treatment trajectories [25]. Outreach medicine should be conceived not as a substitute for stable care, but as a bridge toward it [18,26,29,30,31,32]. Our experience suggests that coordinated action between NGOs and public institutions is feasible and necessary to initiate treatment and to facilitate subsequent transition into institutional pathways.

This study has several limitations. First, many diagnoses were based on clinical assessment in a mobile-clinic setting and were not systematically confirmed by laboratory or instrumental investigations. Second, the analysis is descriptive and cannot establish causal relationships between living conditions and prescription patterns. Third, mental health was not systematically screened, despite the possibility that psychological distress may have contributed to somatic presentations such as fatigue, musculoskeletal pain, and gastrointestinal symptoms [33,34]. Fourth, the data derive from individuals who voluntarily accessed the mobile clinic while living in the informal settlements under study. Migrant workers who did not seek care, had no current complaints, or were excluded from documented care for any reason are not represented in our database. The denominator of our analyses is therefore the set of recorded encounters and therapeutic events, not all migrant workers residing in the settlements, and the findings cannot be used to estimate disease prevalence or incidence rates for the entire population. Finally, the results reflect one regional outreach experience and may not be fully generalizable to other migrant populations or settings.

## 5. Conclusions

This study provides a descriptive account of pharmacological care delivered to migrant agricultural workers living in informal settlements in Apulia. The observed treatment patterns were largely centred on symptomatic management, particularly for musculoskeletal, gastrointestinal, respiratory, dermatological, dental, and cardiovascular conditions.

Although the descriptive design does not allow causal inference, the pharmacological profile observed in this outreach setting may be interpreted as consistent with unmet health needs in a population facing marked social and healthcare vulnerability. These findings support the need for stronger inclusion of migrant agricultural workers in the National Health Service, earlier access to primary care, and improved continuity of care. Outreach services should function as a bridge to institutional healthcare, rather than a substitute for it.

## Figures and Tables

**Table 1:** Frequency of clinical diagnoses and presenting symptoms among the study population. Categories are grouped by organ system or clinical manifestation to highlight the primary reasons for encounter.

Clinical Diagnoses	*N*	%	Presenting Symptoms
Fatigue & Musculoskeletal problems	2907	34.01%	Fatigue Syndrome, Osteo-articular, Asthenia
Gastrointestinal disorders	1157	13.55%	Gastrointestinal, Constipation, Diarrhea, Hepatitis
Airways pathologies	1107	12.95%	Airway infections, tuberculosis, asthma, rhinitis, lung diseases
Dermatological	944	11.05%	Dermatological, Itching, Intertrigo, Parasitosis
Cardiovascular and Metabolic	755	8.84%	Heart problems & Hypertension (651), Diabetes & Endocrine diseases
Dental problems	553	6.47%	Dental, Maxillofacial, Caries
Traumas	378	4.42%	Trauma, Fractures, Wounds, Dressings, Surgery
Ophthalmological	333	3.90%	Ophthalmology, Conjunctivitis, Blepharitis
Urinary tract	136	1.59%	Urinary tract, Renal failure, Venereal diseases
ENT pathologies (ORL)	102	1.19%	Ear, nose, and throat disorders
Neuropsychiatric disorders	91	1.06%	Neurological, Psychiatric, Insomnia, Headaches
Rheumatological	43	0.50%	Rheumatology, Arthritis, Spondylolisthesis
Obstetric-gynecological	25	0.29%	Obstetrics and Gynecology, Pregnancy
Hematological	9	0.1%	Hematology
Oncological	7	0.08%	Neoplasms and carcinomas
Total	8547	100.00%	

**Table 2:** Distribution of prescribed pharmacological treatments by therapeutic class. Data represent total occurrences and percentage frequency of drugs administered during the study period.

Drug Category	*N*	% of Total	Included Items
NSAIDs & Analgesics	2919	32.56%	NSAIDs, Ibuprofen, Paracetamol
Topical Treatments & Dressings	1274	14.22%	Topicals, Wound care, ointments and creams, eye drops
Adjuvants & Supplements	1098	12.25%	Adjuvants, Vitamins, Minerals, Dietary supplements
None/Diagnostics	877	9.76%	No therapy, Negative swabs, Isolation
Gastroenterology	757	8.46%	PPIs & Antacids (6,8%), Laxatives, Probiotics, Antispasmodics, Antidiarrheals
Respiratory System	720	8.03%	Mucolytics, Antihistamines, Bronchodilators, Systemic Corticosteroids
Cardiovascular & Metabolic System	640	7.14%	Antihypertensives, Diuretics, Antidiabetics, Insulin
Antibiotics & Systemic Antifungals	634	7.07%	Oral/Injectable Antibiotics and Antifungals
Other (Neurology, Ophthalmology, etc.)	21	0.23%	Antiepileptics, Antipsychotics
Procedures & Urgencies	25	0.28%	ER referrals, Dialysis, Surgical procedures
TOTAL	8965	100.00%	

**Table 3:** Pharmacological treatments by diagnostic subgroup. Pharmacological treatments by diagnostic subgroup. The ATC classes we considered are associated with each drug category.

Diagnostic Subgroup	Therapeutic Subgroup	ATC Code(s)
Musculoskeletal/fatigue	NSAIDs, analgesics and muscle relaxants	M01, N02, M03
Musculoskeletal/fatigue	Vitamins and supplements	A11, A12
Musculoskeletal/fatigue	Topical treatments/local interventions	M02, D02
Gastrointestinal	Gastroprotective agents	A02
Gastrointestinal	Probiotics and supplements	A07, A11, A12, A07FA
Gastrointestinal	Symptomatic GI drugs	A06, A03, A04, A07XA
Gastrointestinal	Antibiotics	J01
Respiratory	Anti-inflammatory agents	M01, N02
Respiratory	Symptomatic respiratory drugs	R05, R06, R03
Respiratory	Antibiotics	J01
Dermatological	Topical treatments	D01, D02, D03, D04, D06, D07
Dermatological	Oral antihistamines	R06
Dermatological	Oral antifungals	J02
Dermatological	Oral antibiotics/antivirals	J01, J05
Dental	NSAIDs and analgesics	M01, N02
Dental	Antibiotics	J01
Cardiovascular	Antihypertensives/beta-blockers/diuretics	C02, C07, C03, C08, C09
Cardiovascular	Antithrombotic agents	B01

## Data Availability

The NGO “Cuamm-Doctors with Africa,” which is leading the project described in the research, is available to share the data collected during the visits for further reporting and progress. For further information, please contact the corresponding authors.

## Abbreviations

The following abbreviations are used in this manuscript:

CUAMM	Collegio Universitario Aspiranti Medici Missionari (Doctors with Africa)
ENT	Ear, Nose, and Throat (Otorhinolaryngology)
GP	General Practitioner
NCDs	Non-Communicable Diseases
NGO	Non-Governmental Organization
NSAIDs	Non-Steroidal Anti-Inflammatory Drugs
PPIs	Proton Pump Inhibitors
STP	Straniero Temporaneamente Presente (Temporarily Present Foreigner code)
